# Assessment of ventricular flow dynamics by 4D-flow MRI in patients following surgical repair of d-transposition of the great arteries

**DOI:** 10.1007/s00330-021-07813-0

**Published:** 2021-03-30

**Authors:** Fraser M. Callaghan, Barbara Burkhardt, Emanuela R. Valsangiacomo Buechel, Christian J. Kellenberger, Julia Geiger

**Affiliations:** 1 University of Zurich, Zurich, Switzerland; 2 Center for MR-Research, University Children’s Hospital, Steinwiesstrasse 75, 8032 Zurich, Switzerland; 3 Children’s Research Center, University Children’s Hospital, Zurich, Switzerland; 4 Division of Pediatric Cardiology, University Children’s Hospital, Zurich, Switzerland; 5 Department of Diagnostic Imaging, University Children’s Hospital, Zurich, Switzerland

**Keywords:** Transposition of great vessels, Ventricular function, Magnetic resonance imaging, Viscosity

## Abstract

**Objectives:**

To use 4D-flow MRI to describe systemic and non-systemic ventricular flow organisation and energy loss in patients with repaired d-transposition of the great arteries (d-TGA) and normal subjects.

**Methods:**

Pathline tracking of ventricular volumes was performed using 4D-flow MRI data from a 1.5-T GE Discovery MR450 scanner. D-TGA patients following arterial switch (*n* = 17, mean age 14 ± 5 years) and atrial switch (*n* = 15, 35 ± 6 years) procedures were examined and compared with subjects with normal cardiac anatomy and ventricular function (*n* = 12, 12 ± 3 years). Pathlines were classified by their passage through the ventricles as direct flow, retained inflow, delayed ejection flow, and residual volume and visually and quantitatively assessed. Additionally, viscous energy losses (EL_*v*_) were calculated.

**Results:**

In normal subjects, the ventricular flow paths were well ordered following similar trajectories through the ventricles with very little mixing of flow components. The flow paths in all atrial and some arterial switch patients were more irregular with high mixing. Direct flow and delayed ejection flow were decreased in atrial switch patients’ systemic ventricles with a corresponding increase in residual volume compared with normal subjects (*p* = 0.003 and *p* < 0.001 respectively) and arterial switch patients (*p* < 0.0001 and *p* < 0.001 respectively). In non-systemic ventricles, arterial switch patients had increased direct flow and decreased delayed ejection fractions compared to normal (*p* = 0.007 and *p* < 0.001 respectively) and atrial switch patients (*p* = 0.01 and *p* < 0.001 respectively). Regions of high levels of mixing of ventricular flow components showed elevated EL_*v*_.

**Conclusions:**

4D-flow MRI pathline tracking reveals disordered ventricular flow patterns and associated EL_*v*_ in d-TGA patients.

**Key Points:**

*• 4D-flow MRI can be used to assess intraventricular flow dynamics in d-TGA patients.*

*• d-TGA arterial switch patients mostly show intraventricular flow dynamics representative of normal subjects, while atrial switch patients show increased flow disorder and different proportions of intraventricular flow volumes.*

*• Flow disruption and disorder increase viscous energy losses.*

**Supplementary Information:**

The online version contains supplementary material available at 10.1007/s00330-021-07813-0.

## Introduction

Dextro-transposition of the great arteries (d-TGA) is a congenital heart defect characterised by the transposition of the primary arteries exiting the heart resulting in deoxygenated blood being passed to the aorta and returned to the body, while oxygenated blood is returned from the left ventricle to the lungs in a closed circuit. D-TGA accounts for 5–7% of all congenital heart defects and requires surgical intervention to ensure patient survival [1]. Previously, patients have been treated with an atrial switch procedure, redirecting pulmonary blood from the lungs to the right ventricle and thus to the transposed aorta and body; however, this has been gradually replaced by the arterial switch procedure, which transects the aorta and main pulmonary artery, returning them to their natural position and resulting in improved survival rates [2]. With arterial switch operations only surpassing atrial switch corrections in the early 1990s [2], the ongoing evaluation and assessment of surgically corrected d-TGA patients is mandatory to assess mid- and long-term sequelae.

Whereas short-term follow-up is excellent in most of the patients, there are typical potential mid- to long-term complications for both operations [3–6]. Atrial switch patients, after a Senning procedure, face a higher risk of atrial or ventricular arrhythmia as the right ventricle must maintain systemic circulation [3, 4]. This patient cohort also requires regular follow-up imaging by echocardiography and MRI to recognise pathologies that need re-interventions, mostly concerning the right ventricular outflow tract [1, 2, 7, 8].

4D-flow MRI provides a time-dependent, high-resolution velocity vector field, able to be interrogated retrospectively for a number of features such as flow volumes, flow patterns, pressure differences, energy loss, and wall shear stress [9, 10]. The technique is showing valuable promise in the area of congenital heart disease [11] and previous studies using flow-derived parameters such as kinetic energy and viscous energy loss (EL_*v*_) in patients with tetralogy of Fallot or Fontan palliation have shown the potential of 4D-flow MRI for a more comprehensive intraventricular assessment compared to traditional volumetrics [12–15].

4D-flow MRI data in healthy subjects has shown that there are well-defined flow fractions that pass through the ventricles relative to the cardiac cycle [16–18]. These comprise a direct flow fraction, retained inflow, delayed ejection, and residual flow volume. By using this approach, we aim to assess the systemic and non-systemic ventricular dynamics of d-TGA patients following either atrial or arterial switch repair as well as normal subjects. We predict that the flow dynamics in d-TGA subjects will be disturbed and deviate from that of normal subjects which in turn will have implications on intraventricular energetics.

## Methods

### Patients

Patients presenting for routine cardiac MRI assessment between April 2018 and October 2019 were retrospectively included in this study if they provided written, informed consent for retrospective use of their imaging data and if they had a corrected d-TGA (atrial switch repair according to Senning or arterial switch repair) or were found to have normal cardiac function and anatomy following examination.

### Data acquisition

All subjects underwent cardiac MRI examination as part of their clinical assessment. All scanning was performed on a 1.5-T GE Discovery MR450 scanner using a 32 channel cardiac coil. As part of a standard clinical examination, short-axis, four-chamber, two-chamber, and ventricle outflow tract CINE MRI views were obtained, as well as a 4-point 4D-flow acquisition covering the entire chest extending from below the heart inferiorly, to above the origin of the aortic arch branches superiorly. 4D-flow sequences were electrocardiogram gated with free breathing and were performed following a contrast-enhanced agent (Dotarem®; gadoterate meglumine) magnetic resonance angiography sequence (mean time between contrast injection and 4D-flow sequence was 4.4 ± 3.9 min). Acquisitions had axial slice orientation, echo time was 2.4 ± 0.04 ms, repetition time 4.3 ± 0.1 ms, and a flip angle of 15° was used. Velocity encoding ranged from 160 to 200 cm/s dependent on initial 2D phase-contrast acquisitions at the ascending aorta and main pulmonary artery. Spatial resolution was acquired at a range of 1.3–2.7 mm in-plane resolution (median 2.0 mm), reconstructed to 0.9–1.6 mm (median 1.4) and slice thickness ranged from 1.8 to 2.7 mm (median 2.0 mm). The mean temporal resolution was 33.9 ± 9.2 ms equating to 20–25 phases per cardiac cycle. A local shim volume covering the heart and major vessels was defined, segmentation factor of three was used and a k-t acceleration parallel imaging technique (*kat-ARC* = 8) was employed [19].

### Data analysis—volumetry

Using four-chamber, two-chamber, and outflow tract views for correct orientation, ventricle lumen contours were drawn at end-diastolic and end-systolic time points on the short-axis images for both ventricles using a semi-automated technique in the Medis QMass software (version 8.1). The resulting end-diastolic volume (EDV), stroke volume (SV), and ejection fraction (EF) were calculated. Unless otherwise specified, EDV and SV are reported indexed by body surface area (BSA).

### Data analysis—4D-flow post processing

4D-flow MRI data were inspected manually for phase aliasing and data quality (an absence of patient movement or significant wrap around or ghosting artefact) and were corrected for background phase offsets using a 4^th^-order polynomial fit to static tissue. Mitral (MV) and tricuspid valve (TV) annulus planes were marked and tracked over time manually using four-chamber CINE scans. At each 4D-flow acquisition time step, the valve orifice was manually drawn on a reformatted 4D-flow image at the level of the corresponding MV and TV annulus plane. At the aortic and pulmonary valves, ROIs were manually drawn tracking the artery movements over time using just the 4D-flow data. To avoid confusion, ventricles are classified as systemic or non-systemic ventricles, depending on the circulation that they supply.

Pathline tracking was performed using the scientific visualisation software ParaView (Kitware Inc.). Source points were defined over an isotropic domain covering the end-diastolic ventricular volume but not crossing the inlet or outlet ROIs. The particle points were tracked both forwards and backwards over time starting from end-diastole, for one cardiac cycle using a 4^th^-order Runge-Kutta integration scheme. Particles that passed through the inlet ROI were considered to have originated from the left atrium or pulmonary veins (in the case of the systemic ventricle), while particles that crossed the outlet ROI were considered to be ejected from the ventricle during systole. Thus, particles that originated from the same source point and passed through the inlet ROI during backward time tracking and passed through the outlet ROI during forward time tracking were classified as direct flow according to the definitions of Eriksson et al [16]. Similarly, retained inflow—those entering the ventricle during diastole but not being ejected during the following systolic phase; delayed ejection—those residing within the ventricle at end-diastole and exiting during ejection; and residual volume—those residing in the ventricle at end-diastole and not being assigned to one of the other classifications were also identified. The accuracy of the flow particle classification was evaluated by comparing ventricle inflow and outflow volumes and by comparing the ejected volume independently against the stroke volume calculated from the short-axis volumetry. The path of travel of particles was assessed visually with respect to their classifications.

In addition, total EL_*v*_ was calculated over the cardiac cycle. EL_*v*_ are irreversible losses to thermal energy resulting from the frictional (viscous) interaction of fluid flow with itself and its boundaries. The instantaneous rate of EL_*v*_ can be calculated from the 3D velocity field (*ν*) as:

1 $$ {\phi}_v=\frac{1}{2}{\sum}_i{\sum}_j{\left[\left(\frac{\partial {v}_i}{\partial {x}_j}+\frac{\partial {v}_j}{\partial {x}_i}\right)-\frac{2}{3}\left(\nabla \cdotp v\right){\partial}_{ij}\right]}^2 $$

where *i* and *j* describe principal directions in the domain *x* and *∂*_*ij*_ is the Kronecker delta. The rate of energy loss within the ventricle is the product of blood viscosity, assumed constant at 3.5 × 10^−3^ *Pa* ∙ *s*, and the summation of *ϕ*_*v*_ over the ventricle volume. Total energy loss, measured in Joules, over a cardiac cycle is calculated by the integration of the rate of energy loss over time [20]. Given the variability in the study cohort, a normalised, by end-diastolic volume, total viscous energy loss ($\overline{EL_v}$) was also calculated.

### Statistical analysis

Data analysis was performed using the public environment for statistical computing R (version 3.2.3). Unless specified, data are expressed as mean ± one standard deviation. Data for each flow classification component (direct flow, delayed ejection, and retained inflow) and each ventricle (systemic or non-systemic) were tested for normality via the Shapiro-Wilk test and compared with one-way ANOVA and then between arterial and atrial switch d-TGA groups and normal subjects using Student’s *t* test. Significance is defined at a level of *p* < 0.05.

## Results

### Study cohort and ventricle volumetrics

**Fig. 1:**
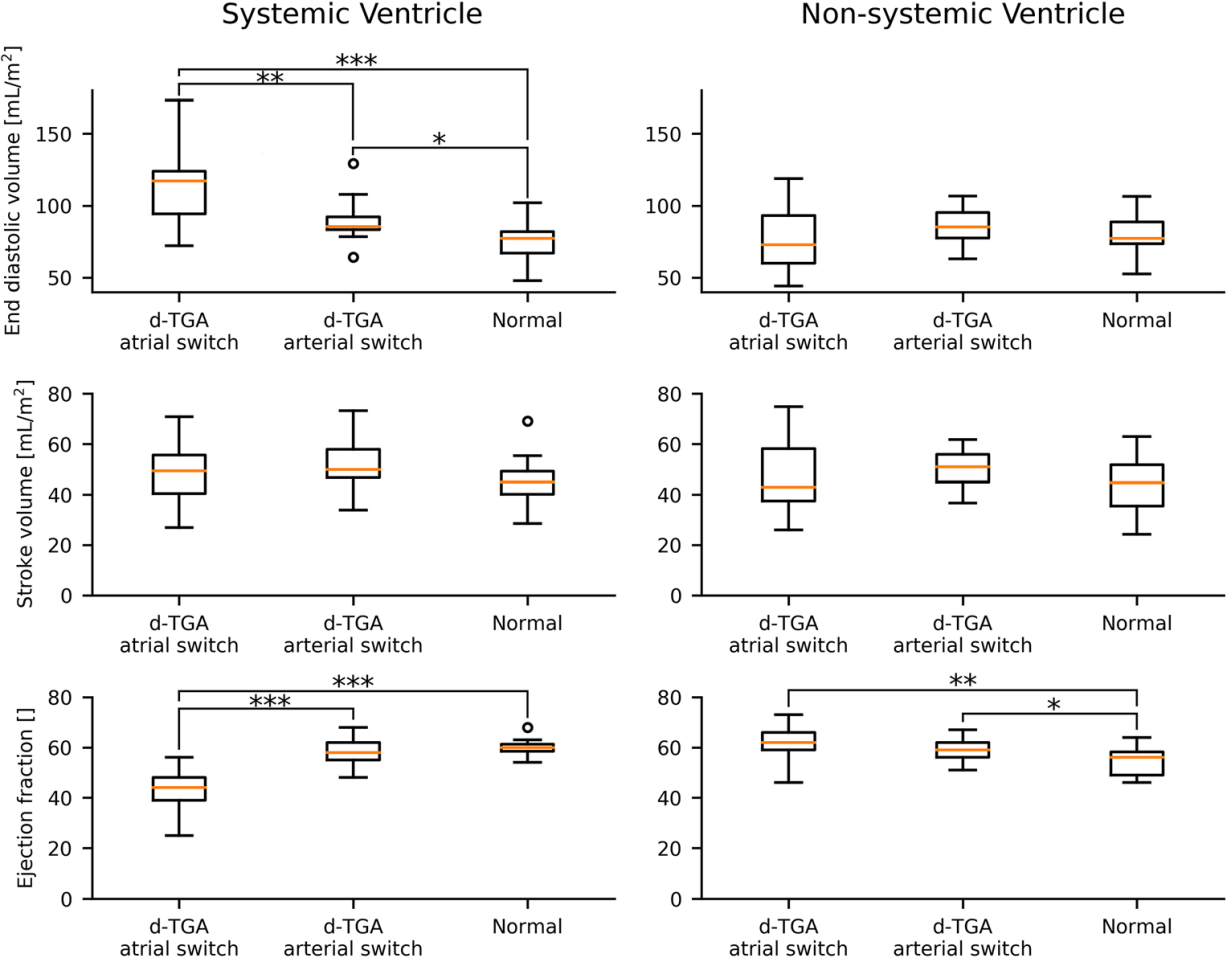
Box and whisker plots showing the distribution of (BSA normalised) end-diastolic volume, stroke volume, and ejection fraction in the systemic (left column), and non-systemic (right column) ventricles of d-TGA atrial switch and arterial switch patients and normal subjects. Boxes depict median (middle bar), upper and lower quartile values. Outliers are data 1.5x the inter-quartile range and are indicated with dots. Significant differences between cohort measures are indicated by *p < 0.05; **p < 0.01; ***p < 0.001

The study included 15 d-TGA patients following atrial switch repair, 17 d-TGA patients after arterial switch procedure, and 12 patients with normal cardiovascular anatomy and function. One subject from each cohort group was rejected for poor quality short-axis cine data that prevented ventricle segmentation. One additional arterial switch subject was excluded due to excessive aortic insufficiency. All included subjects had good-quality 4D-flow MRI data and quality control measures confirmed pathline volume fractions to within 13% agreement with ventricular stroke volumes. Full demographic and ventricle volumetric details are provided in Table 1 and illustrated in Fig. 1. Importantly, atrial switch patients were significantly older and had higher BSA values (*p* < 0.001 for both) than both arterial switch and normal anatomy patients, whereas age and BSA were similar between normal and arterial switch patients. Atrial switch subjects had significantly larger, normalised, systemic ventricle EDV and lower systemic ventricle EF compared with patients after arterial switch procedure and those with normal anatomy. In the non-systemic ventricle, EF was slightly higher in both atrial and arterial switch patients compared to normal patients (*p* = 0.007 and 0.039 respectively).

**Table 1:** Demographics and normalised ventricular volume measurements

				*p* values		
	Atrial switch	Arterial switch	Normal	Atrial vs arterial switch	Normal vs atrial switch	Normal vs arterial switch
*N*	15	17	12			
Gender M/F (%M)	10/5 (67%)	11/6 (65%)	8/4 (67%)			
Age [yr]	35 ± 6	14 ± 5	12 ± 3	< 0.0001	< 0.0001	0.18
BSA [m^2^]	1.82 ± 0.16	1.41 ± 0.4	1.42 ± 0.3	0.001	0.0006	0.99
Volumetrics						
Systemic ventricle						
EDV [mL/m^2^]	112.7 ± 27.6	89.3 ± 13.9	76.2 ± 14.9	0.004	0.0003	0.02
SV [mL/m^2^]	48.0 ± 13.2	51.6 ± 9.5	45.2 ± 10.3	0.38	0.59	0.12
EF [%]	42.9 ± 7.4	57.9 ± 5.8	59.1 ± 4.7	< 0.0001	< 0.0001	0.31
Non-systemic ventricle						
EDV [mL/m^2^]	76.2 ± 20.9	85.8 ± 13.0	82.9 ± 18.6	0.13	0.60	0.31
SV [mL/m^2^]	47.5 ± 14.8	50.5 ± 7.2	44.4 ± 10.6	0.46	0.52	0.069
EF [%]	62.0 ± 6.6	59.1 ± 4.6	53.8 ± 6.9	0.16	0.007	0.039

### Visual analysis of ventricle flow dynamics

**Fig. 2:**
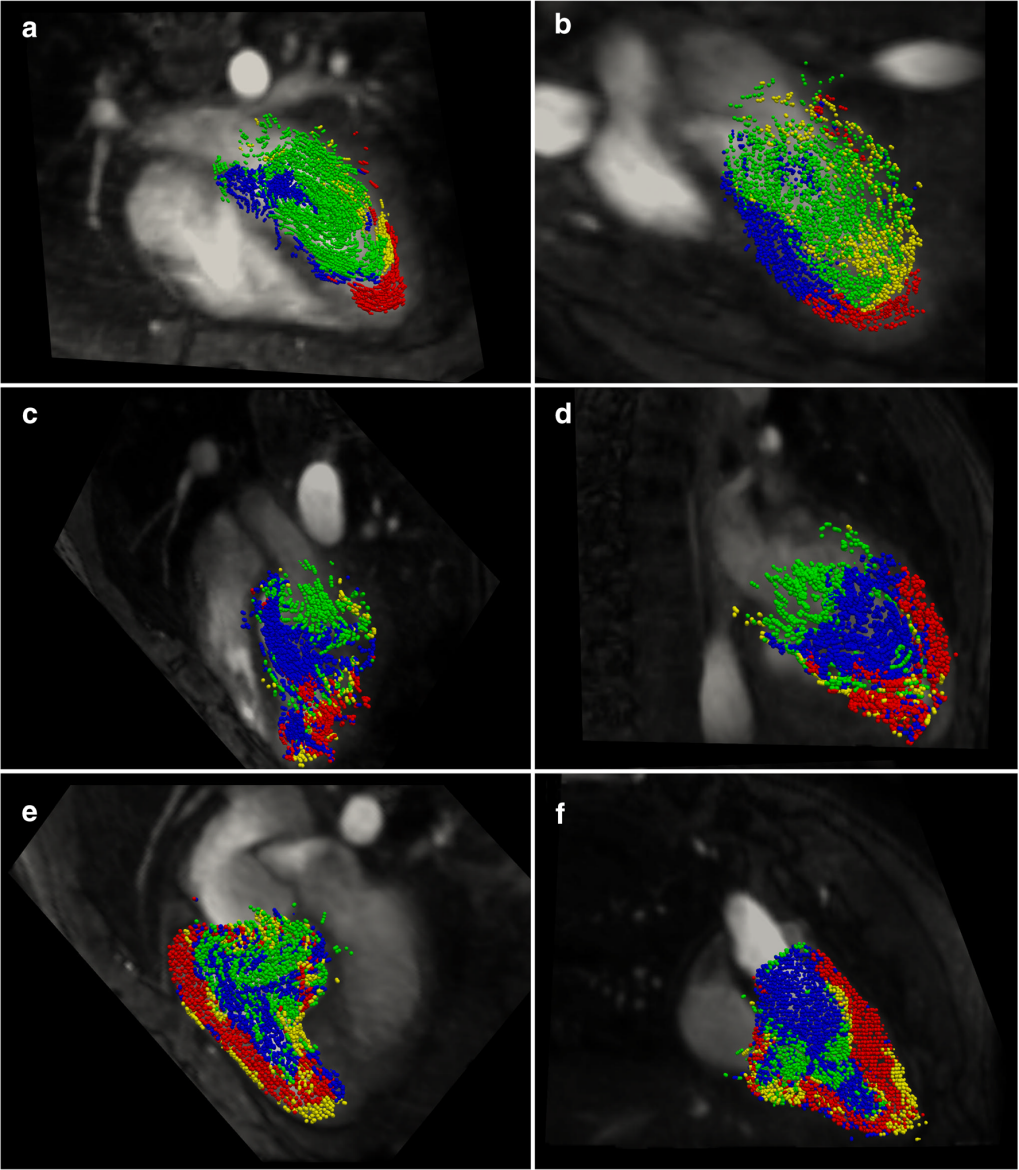
The location of systemic ventricular blood volumes at the time point of end-diastole on two orthogonal views in a representative normal (**a** and **b**), d-TGA arterial switch (**c** and **d**), and a d-TGA atrial switch patient (**e** and **f**). Views **a**, **c**, and **e** show a four-chamber view and views **b**, **d**, and **f** show an orthogonal plane passing through the apex and systemic outflow tract. The illustrated blood volumes are restricted to the plane of interest and are classified as direct flow (green), delayed ejection (blue), retained inflow (yellow), and residual volume (red)

Visually, the flow dynamics followed an ordered and distinctly structured profile for all normal subjects. In the systemic ventricle, the direct flow and retained inflow portions entered and moved towards the apex, inducing circulation in a path from the mitral valve towards the apex and then following the septal wall towards the outflow tract, driving the ventricle volumes already present at end-systole (delayed ejection and residual volume) in this direction. The direct flow portion was more centrally orientated and continued following this path to be ejected during the following systolic phase. The retained inflow portion moved along the ventricle free-wall and was retained in the ventricle during the following systolic phase. The delayed ejection portion filled the outflow tract and posterior-septal wall region of the ventricle at end-diastole and was ejected during systole. Figure 2a and b show the distribution of flow portions at end-diastole for a normal subject.

**Fig. 3:**
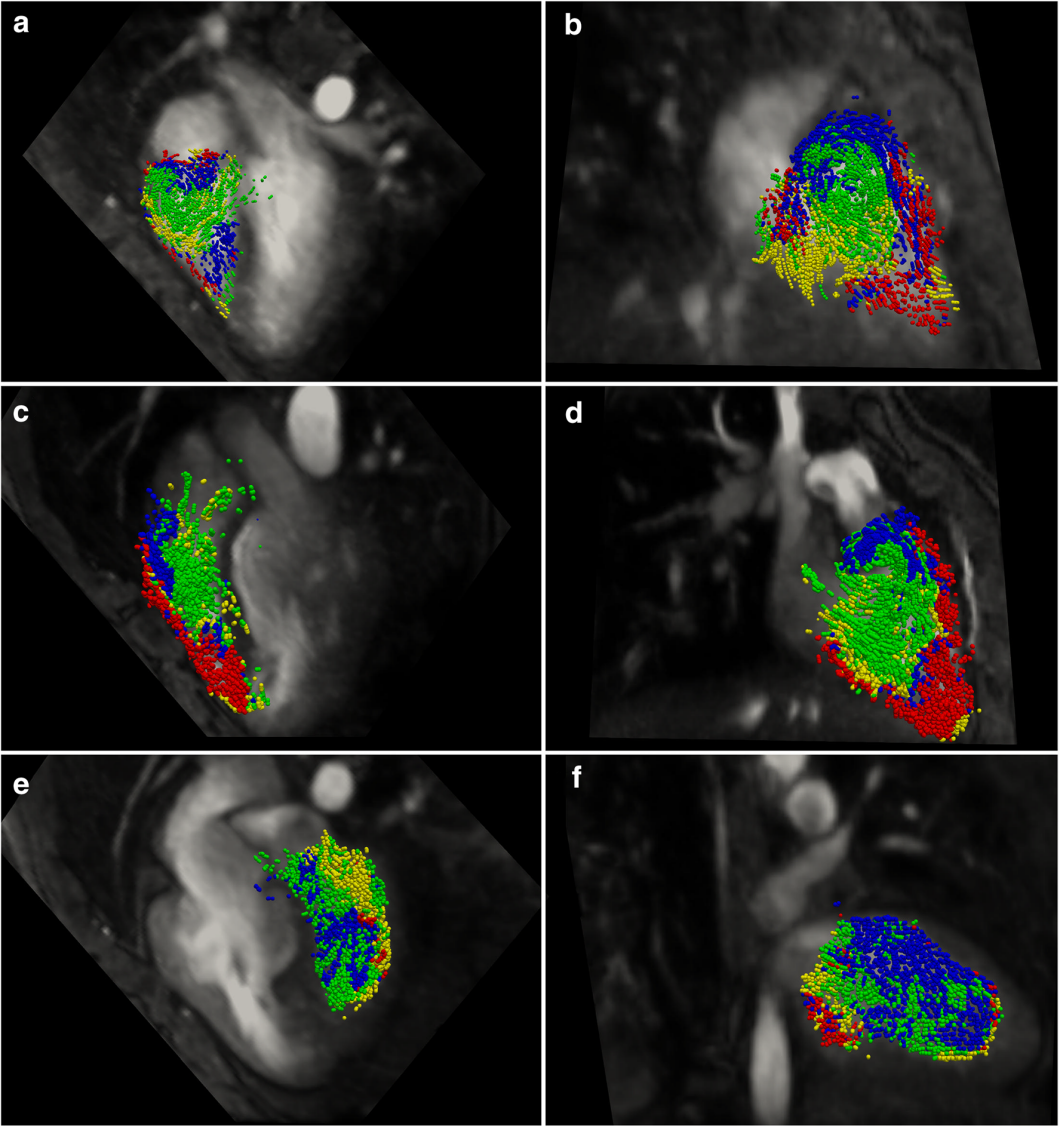
The location of non-systemic ventricular blood volumes at the time point of end-diastole on two orthogonal views in a representative normal (**a** and **b**), d-TGA arterial switch (**c** and **d**), and a d-TGA atrial switch patient (**e** and **f**). Views **a**, **c**, and **e** show a four-chamber view and views **b**, **d**, and **f** show an orthogonal plane passing through the apex and outflow tract. The illustrated blood volumes are restricted to the plane of interest and are classified as direct flow (green), delayed ejection (blue), retained inflow (yellow), and residual volume (red)

In the non-systemic ventricle, the retained inflow appeared to diverge into the various trabeculations which had been compressed at end-systole and the direct flow portion made a very direct path from tricuspid valve to pulmonary valve (Fig. 3a and b).

In arterial switch patients, the flow patterns in the systemic and non-systemic ventricles typically mimicked that of the normal subjects. In subjects who showed dissimilar flow patterns, increased mixing between the direct flow and delayed ejection components was obvious at end-diastole, which were then ejected together over systole (Figs. 2c, d and 3c, d).

Atrial switch patients showed variable flow dynamics which deviated from those observed in both the systemic ventricles and non-systemic ventricles of the normal subjects. There was a higher degree of mixing between the components (Figs. 2e, f and 3e, f). While Figs. 2 and 3 demonstrate a temporal snapshot of the flow order and disorder in representative subjects, Supplementary Movie 1 provides clear animations of the full dynamics for three illustrative subjects.

### Quantification of ventricle flow component volumes

**Fig. 4:**
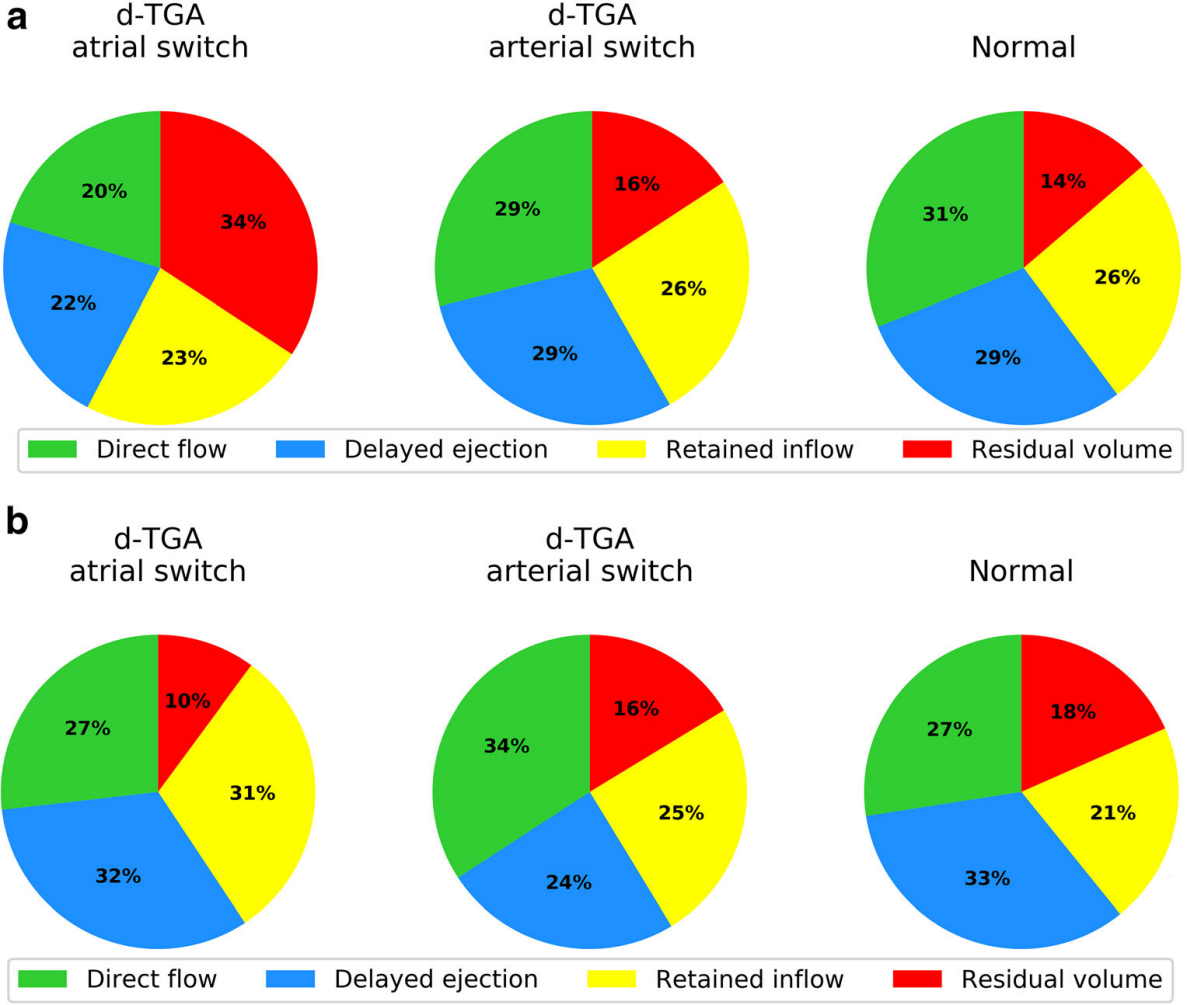
Pie chart representation of ventricular flow components of the systemic ventricle in d-TGA patients (atrial and arterial switch corrections) and normal subjects (upper panel) and the non-systemic ventricle (lower panel)

The fractions of systemic ventricular flow passing as direct flow and as delayed ejection were significantly lower in atrial switch patients compared with those in both arterial switch patients (*p* < 0.0001 and *p* < 0.001 respectively) and normal subjects (*p* = 0.003 and *p* < 0.001 respectively). This was compensated by a significantly larger proportion of residual volume in atrial switch patients compared to arterial switch patients and normal subjects (*p* < 0.0001 for both). Retained inflow fractions were not different. There were no differences between any flow fractions in arterial switch patients compared to normal subjects (Fig. 4a).

In the non-systemic ventricle, the retained inflow fraction in atrial switch patients was significantly higher than that in normal subjects (*p* = 0.02) and arterial switch patients (*p* < 0.001). Residual volume fractions were lower in atrial switch subjects compared to those in normal subjects (*p* = 0.02). The direct flow portion was significantly higher in arterial switch patients compared to both normal subjects (*p* = 0.007) and atrial switch patients (*p* = 0.01). Delayed ejection fractions were significantly lower in arterial switch patients compared to those in both normal subjects (*p* < 0.001) and atrial switch patients (*p* < 0.001) (Fig. 4b).

### Viscous energy losses

**Fig. 5:**
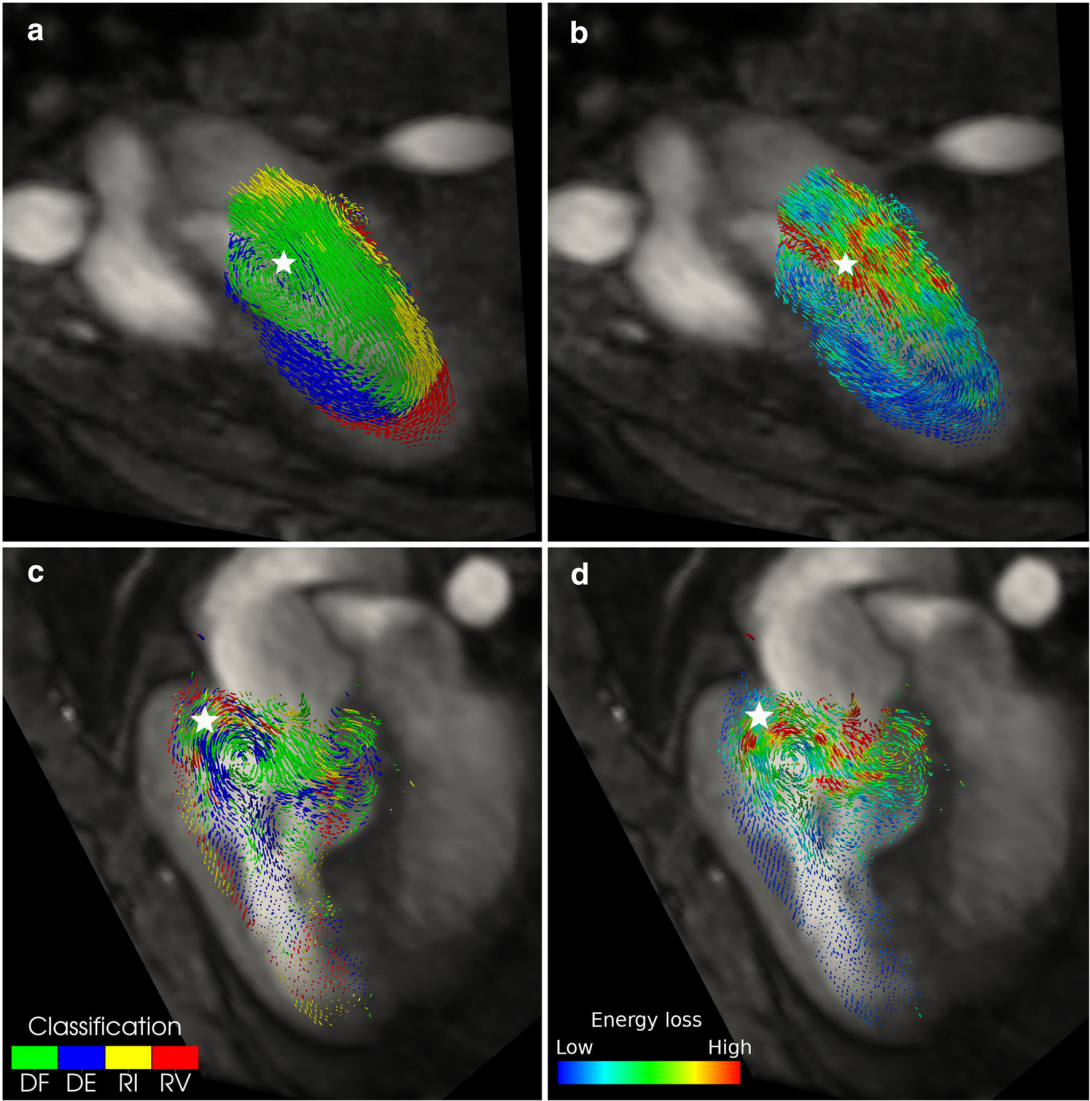
Example of correlation between mixing between flow components and high rates of viscous energy loss in an illustrative normal subject (top row) and a d-TGA patient following atrial switch repair (bottom row). All panels show a snapshot of pathlines upon a plane of the systemic ventricle at approximately end-diastole. Panels **a** and **c** show the pathlines coloured by their respective classification as direct flow (DF), delayed ejection (DE), retained inflow (RI), and residual volume (RV). Panels **b** and **d** show the same image, but with pathlines coloured by the relative magnitude of the rate of viscous energy loss. White stars in each panel indicate locations of mixing of DF and DE components and corresponding high rates of viscous energy loss

Viscous energy losses showed large variability within all three subject groups (Table 2). EL_*v*_ and $\overline{EL_v}$ were significantly higher in the non-systemic ventricles of atrial switch subjects, compared to normal subjects (*p* = 0.03 for both). $\overline{EL_v}$ was significantly decreased in the systemic ventricle of atrial switch patients compared to that of both normal and arterial switch subjects (*p* = 0.01 and 0.005 respectively). There were no significant differences of EL_*v*_ or $\overline{EL_v}$ between normal subjects and arterial switch patients. It was visually observed that subjects with a high EL_*v*_ displayed greater disorder of the ventricle flow classifications. Additionally, regions of high rates of EL_*v*_ tended to correspond to locations of high mixing between separate flow components (Fig. 5).

**Table 2:** Intraventricular viscous energy losses in d-TGA subjects following atrial or arterial switch operations and normal subjects

	Atrial switch	Arterial switch	Normal	Atrial vs jarterial switch	Normal vs atrial switch	Normal vs arterial switch
Viscous energy loss—EL_*v*_ [mJ]						
Systemic ventricle	0.11 ± 0.07	0.12 ± 0.07	0.08 ± 0.05	0.62	0.30	0.11
Non-systemic ventricle	0.13 ± 0.09	0.09 ± 0.06	0.06 ± 0.04	0.17	0.03	0.21
Normalised viscous energy loss—$\overline{E{L}_v}$ [mJ/m^3^]						
Systemic ventricle	527 ± 232	993 ± 560	778 ± 240	0.005	0.01	0.22
Non-systemic ventricle	900 ± 493	724 ± 403	547 ± 194	0.28	0.03	0.17

## Discussion

This study investigated the intraventricular flow volumes and dynamics in d-TGA patients after surgical repair of either atrial switch operation (Senning procedure) or arterial switch operation, in comparison with patients having normal cardiovascular anatomy. We assessed ventricle volumetrics (EDV, SV, and EF) derived from cine short-axis stack MRI data and intraventricular flow classification using 4D-flow MRI. The intraventricular flow classification highlighted well-organised flow patterns in normal paediatric subjects, while varying levels of disorder were found in the arterial switch subjects and high disorder was observed in the flow of adult atrial switch subjects. Arterial switch patients showed a systemic ventricle behaving similarly to normal subjects and an increased proportion of non-systemic ventricle direct flow fraction. Visually, mixing of ventricular flow components corresponded to areas of high rates of viscous energy loss, which was elevated in atrial switch subjects.

Ventricle volumetrics showed expected results concordant with other studies. Normal subjects had EDV, SV, and EF measurements in agreement with published data for children [21]. Arterial switch subjects displayed larger systemic EDV and non-systemic EF values compared to normal subjects in our study; however, these numbers were within normal limits for children [21]. The atrial switch patients in our study had significantly larger systemic EDV volumes and smaller EF compared with both normal and arterial switch subjects. These values were in agreement with previously published data [22]. In the non-systemic ventricle, atrial switch patients had no difference in EDV and SV compared with normal and arterial switch subjects and slightly larger EF compared to normal subjects, also in agreement with previously published results [23].

All normal subjects showed well-structured flow through the ventricles, matching that described in previous studies [17, 18, 24–26]. Atrial switch patients showed a significant decrease in direct flow fraction in the systemic ventricle. This is the morphological right ventricle for these subjects, many of whom had high end-diastolic volumes and low ejection fractions. Thus the reduced direct flow fractions are expected in this scenario. Decreased direct flow and increased residual volume portions have previously been associated with increased EDV and reduced EF values [27]. In the non-systemic ventricle, flow portions of atrial switch patients were more similar to those of the systemic ventricle of normal subjects illustrating that the morphological left ventricle continues to behave representative of a systemic ventricle in atrial switch subjects. Both the transposed ventricles and disturbed inflows, due to redirection via baffles within the atria likely contribute to the differences observed. Arterial switch patients showed elevated direct flow fractions and a decreased delayed ejection fraction compared to normal subjects in the non-systemic ventricle. This may be a result of the slightly different geometry of the outflow tract and orientation of the main pulmonary artery, which is a common outcome following surgery for these subjects [28].

We observed clear flow disorder in some arterial switch and most atrial switch d-TGA subjects, illustrated by mixing of the ventricular flow components, in distinct contrast to the ordered flow patterns observed in all normal, paediatric, subjects and represented in previous publications including healthy adult subjects [18, 25]. Local areas of mixing, especially between flow components that were separate at end-systole, such as the direct flow and delayed ejection portions, corresponded to higher rates of EL_*v*_. Note that the high end-diastolic volumes of the systemic ventricles of atrial switch subjects resulted in low normalised viscous energy losses where high absolute viscous energy losses were observed. Our results are in agreement with other studies that have reported elevated viscous energy loss in disrupted flow dynamics [20, 29–31]. The intraventricular dynamics observed in normal subjects were highly structured and matched those previously described [17, 18, 24, 26]. The inflow volume replaces the delayed/residual volume and drives these volumes towards the outflow tract in preparation for ejection during the following cycle. In the systemic ventricles of subjects showing flow disturbance and mixing of components, the most obvious difference was a disruption of the inlet to apex to the outlet flow path. As a result, flow particles inevitably move “against the flow” and induce friction, which is quantified as viscous energy loss. Elevated viscous energy losses in the presence of disturbance of normal intraventricular flow patterns, specifically, altered vortex ring formation, which may be analogous to the disorganised ventricular flow patterns have been previously described [30].

Other studies have investigated kinetic energy levels in congenital heart disease cohorts, especially patients after Tetralogy of Fallot repair [12–14]. We choose not to pursue this route of investigation preferring to focus on a marker of absolute inefficiency represented by viscous energy loss. This is an irreversible conversion of kinetic to thermal energy due to viscous interactions and has shown promising results in investigations involving disturbed flow dynamics [15, 20, 32, 33]. In patients after Fontan palliation, for example, viscous energy loss was significantly elevated compared to controls, predominantly in patients with discordant inflow versus outflow underlying the impact of structured flow paths and ventricular performance [15].

### Limitations

This study examined three distinct groups: normal anatomy subjects and subjects with d-TGA corrected by arterial or atrial switch repair. The large age difference between the atrial switch group and the other two groups, as well as the significantly different anatomy and relatively low subject numbers limit the impact of inter-group comparisons. The normal anatomy and arterial switch group covered age ranges representing high growth and development [21]. This may explain the disagreement with previously published ventricle flow classification proportions [34]. A recent study covering a similar age range as the current study gives similar left ventricle flow proportions for normal subjects [27], supporting our results and suggesting an age-related change in ventricular haemodynamics.

Some limitations in the accuracy of the techniques applied are to be expected. Using a free-breathing protocol without respiratory gating is a necessity of our clinical practice and this may result in inaccuracies for pathline tracking; however, quality control comparisons of pathline-based stroke volumes compared with volumetric-based stroke volumes suggest this was not critical.

The observations made with regard to the degree of flow component mixing as well as the correlation of this with viscous energy losses are qualitative ones and have not been made under a rigorous verification or quantification process. These results deserve further exploration in a dedicated study, though it is acknowledged that repeatable quantification of such dynamics is difficult.

This study involves an advanced analysis of 4D-flow data, with high user input and long processing time, which is not readily available at a clinical level. The measurement of viscous energy loss is a parameter that relies on the derivative of the velocity field and thus is subject to amplification of errors that may be present due to noise. A typical technique for reducing noise by minimising the encoding velocity was not adopted in this study [35]. Additionally, viscous energy loss measures are dependent on spatial resolution and are known to be underestimated in areas of turbulence, which may be present in the mixing zones observed [36].

## Conclusions

4D-flow MRI identifies both quantitative and qualitative differences in ventricular flow patterns in arterial and atrial switch d-TGA patients and normal subjects. Arterial switch patients generally show flow behaviour and quantification similar to normal subjects. Atrial switch patients displayed disordered ventricular flow compared with the younger cohort of normal subjects in our study and with previously published normal data. Flow disorder in the ventricle leads to increased viscous energy losses.

## Supplementary information

ESM 1 (MP4 5007 kb)

## Abbreviations

$\overline{EL_v}$: Normalised viscous energy loss (by EDV)
4D-flow MRI: Four-dimensional flow MRI
BSA: Body surface area
d-TGA: d-Transposition of the great arteries
EDV: End diastolic volume
EF: Ejection fraction
EL_*v*_: Viscous energy loss
MV: Mitral valve
SV: Stroke volume
TV: Tricuspid valve

## Declarations

### Guarantor

The scientific guarantor of this publication is FMC.

### Statistics and biometry

No complex statistical methods were necessary for this paper.

### Methodology

• retrospective

• observational

• performed at one institution
